# Supplementary figures and images for: Influence of chitosan and chitosan oligosaccharide on dual antibiotic-loaded bone cement: In vitro evaluations
Source: PLoS One. 2022 Nov 30;17(11):e0276604. doi: 10.1371/journal.pone.0276604 (PMC9710798; doi:10.1371/journal.pone.0276604)

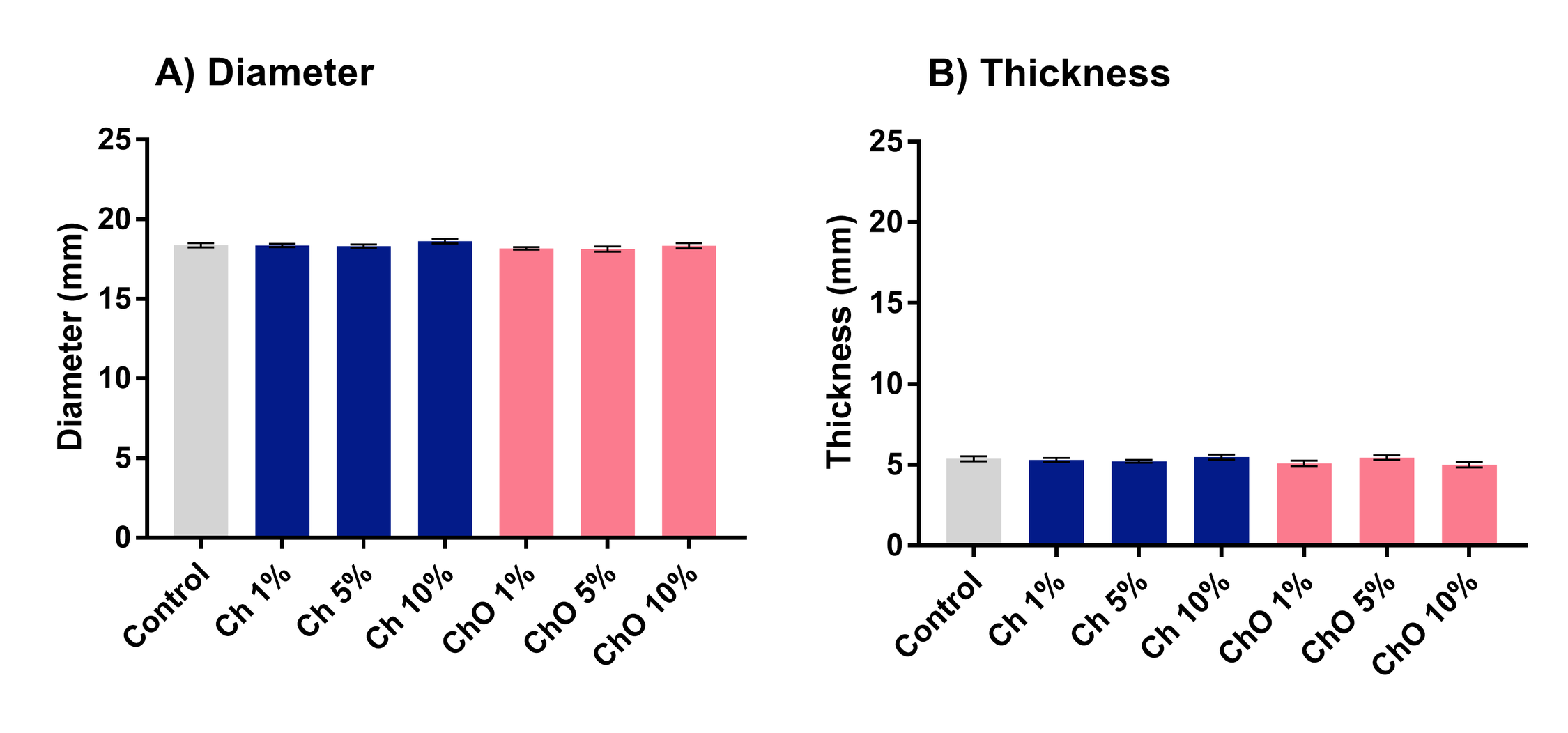

Supplement: S1 Fig — Diameter (A) and thickness (B) of bone cement samples obtained after casting bone cement mixtures in the mold. Control represents bone cement specimens made solely from Copal® G+V. Ch 1%, Ch 5%, and Ch 10% are specimens made of Copal® G+V mixed with 1%, 5% and 10% w/w chitosan, respectively. ChO 1%, ChO 5%, and ChO 10% are specimens made of Copal® G+V mixed with 1%, 5% and 10% w/w chitosan oligosaccharides, respectively. Data are expressed as mean ± SEM (n = 12). One-way ANOVA with Dunnett’s multiple comparisons test was performed. (TIF) [file pone.0276604.s001.tif]

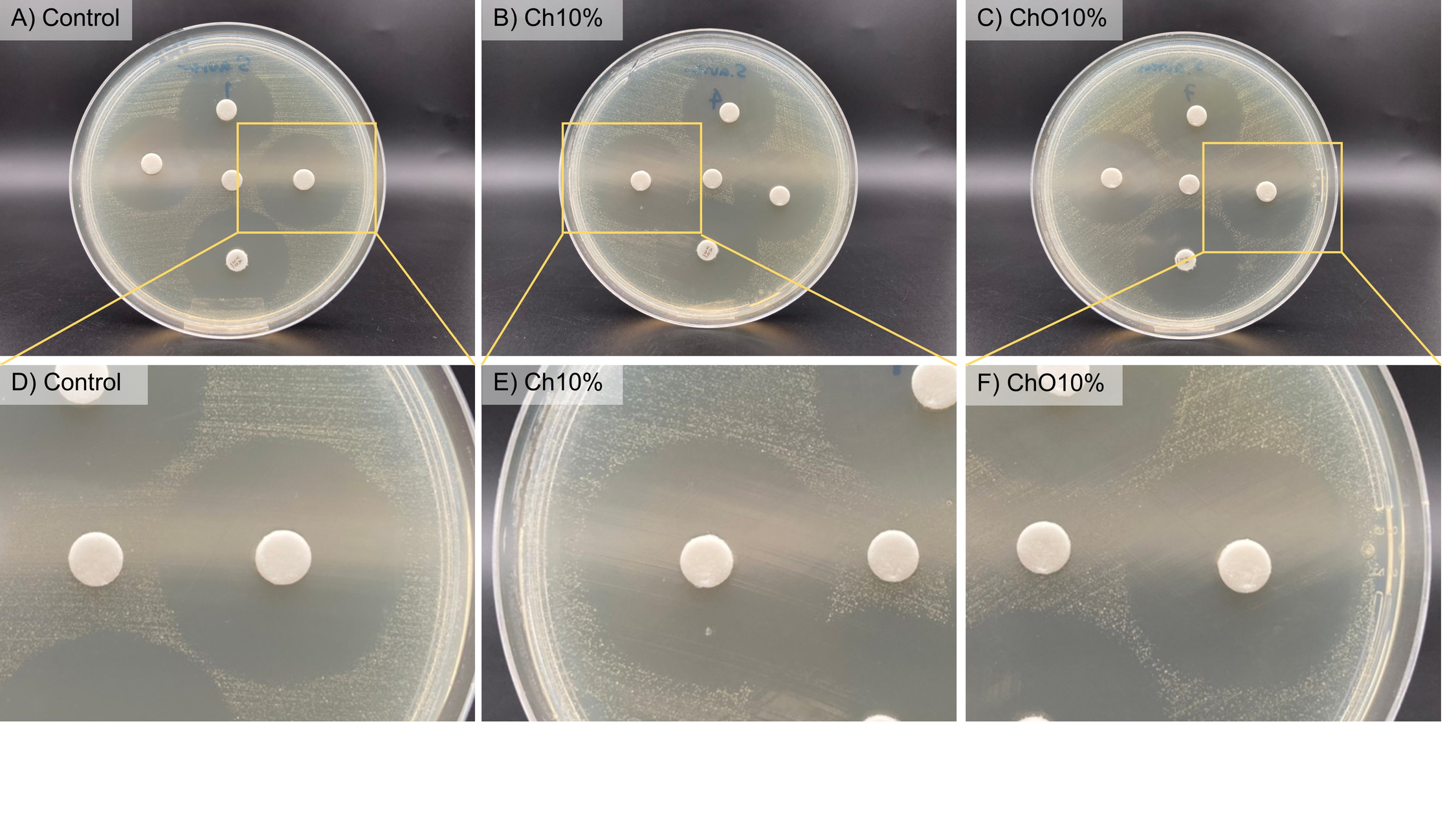

Supplement: S2 Fig — Zone of inhibition (ZOI) of S. aureus was measured. Control represents supernatant obtained from bone cement specimens made solely from Copal® G+V (A, D). Ch 10% are supernatant obtained from specimens made of Copal® G+V mixed with 10% w/w chitosan (B, E). ChO 10% are supernatant obtained from specimens made of Copal® G+V mixed with 10% w/w chitosan oligosaccharides (C, F). (TIF) [file pone.0276604.s002.tif]

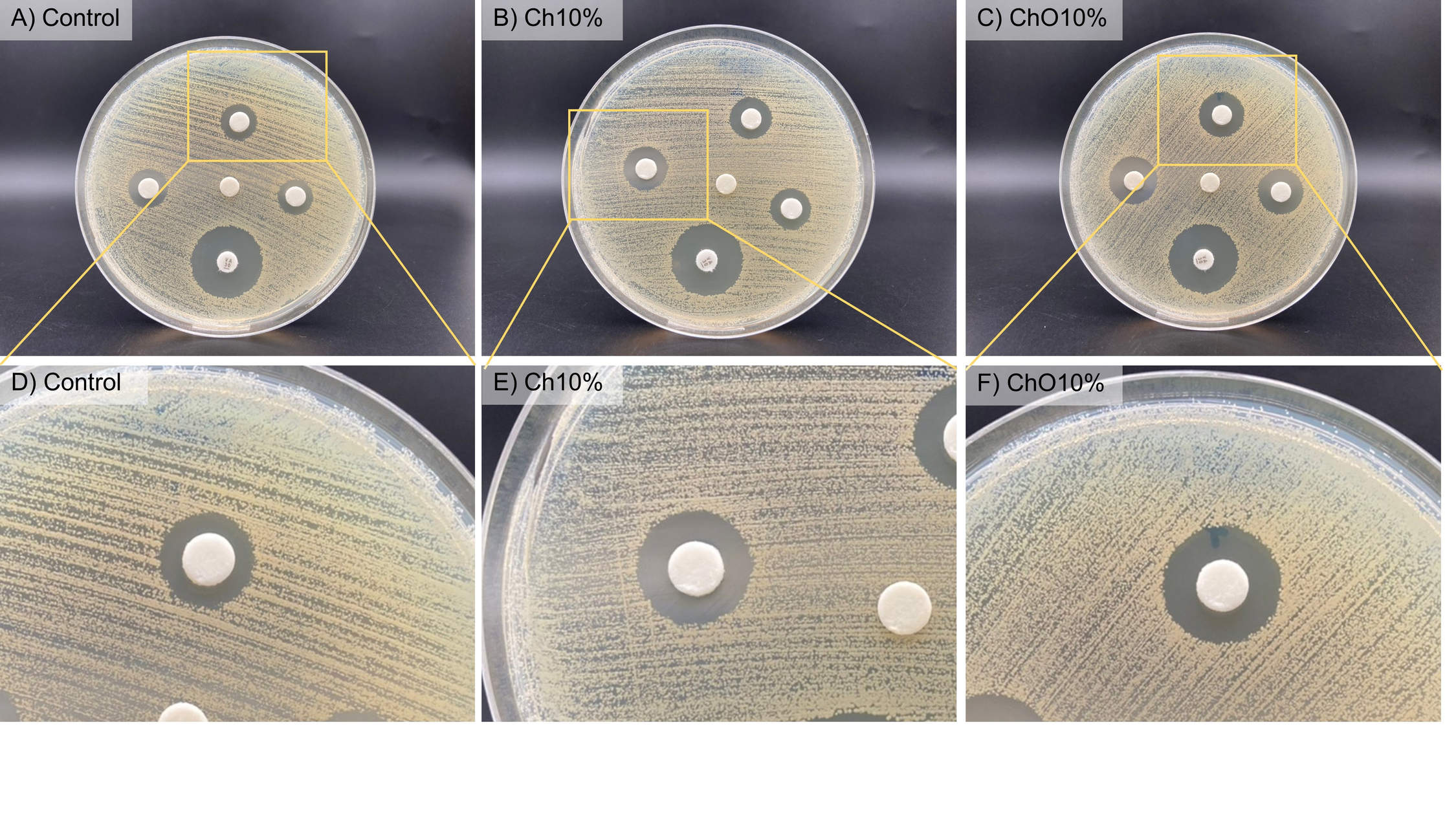

Supplement: S3 Fig — Zone of inhibition (ZOI) of MRSA was measured. Control represents supernatant obtained from bone cement specimens made solely from Copal® G+V (A, D). Ch 10% are supernatant obtained from specimens made of Copal® G+V mixed with 10% w/w chitosan (B, E). ChO 10% are supernatant obtained from specimens made of Copal® G+V mixed with 10% w/w chitosan oligosaccharides (C, F). (TIF) [file pone.0276604.s003.tif]

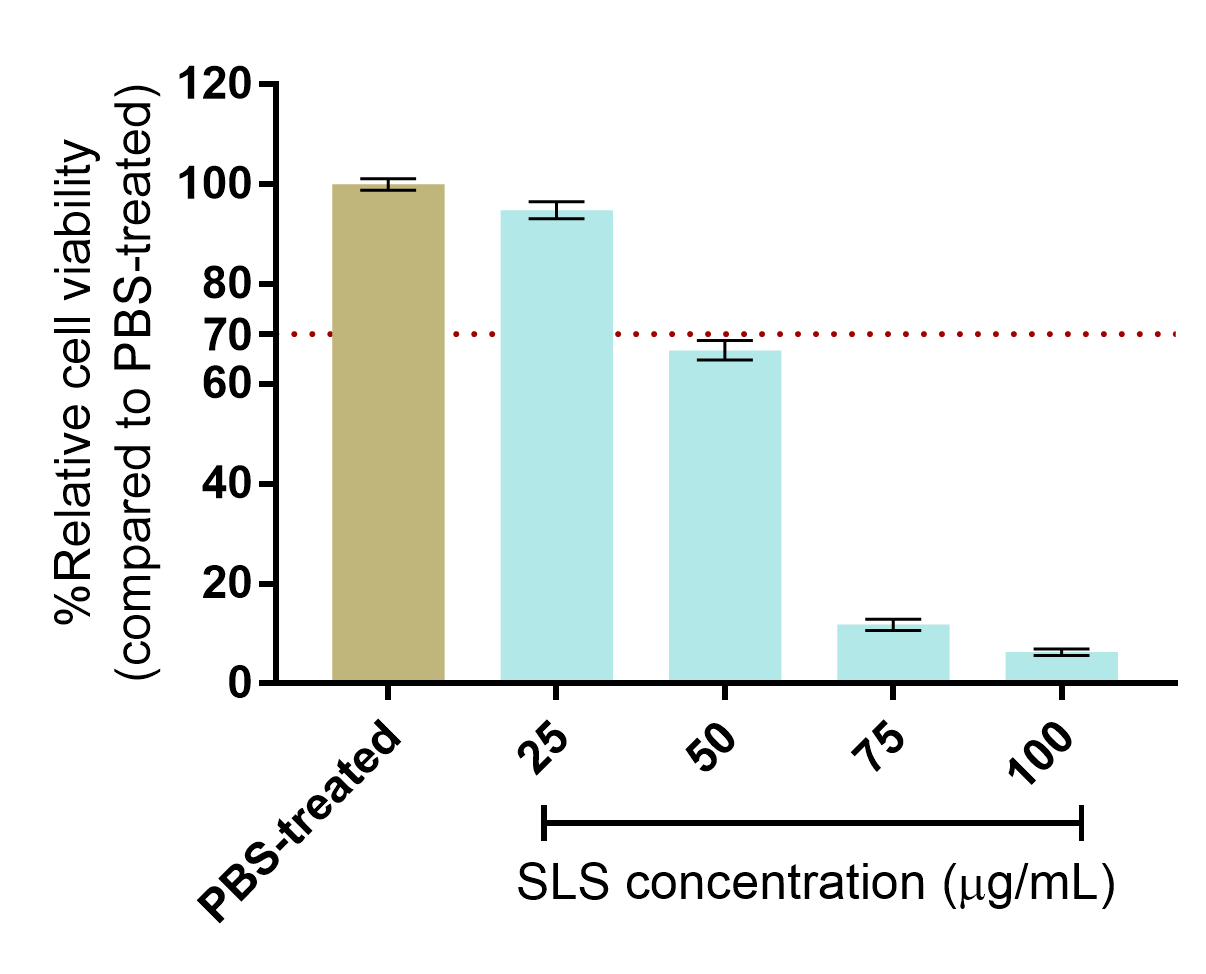

Supplement: S4 Fig — PBS-treated group was considered 100% cell viability. Data are expressed as mean ± SEM (n = 21–42). (TIF) [file pone.0276604.s004.tif]
