# Supplementary material for: Influence of chitosan and chitosan oligosaccharide on dual antibiotic-loaded bone cement: In vitro evaluations
Source: PLoS One. 2022 Nov 30;17(11):e0276604. doi: 10.1371/journal.pone.0276604 (PMC9710798; doi:10.1371/journal.pone.0276604)
Supplement: S1 Table — (PDF) [file pone.0276604.s005.pdf]

Supplementary Table 1 The minimal data set underlying the results

Figure number/Sample name (group)	Sample name (subgroup)	Mean	SEM	Statistical method used	P-value	Sample size (n)	Raw data					
**Figure 3:** Weight (g) of bone cements obtained after casting bone cement mixtures before and after drug elusion												
Before the release study	Control	1.435	0.028	Two-way ANOVA with Dunnett's multiple comparisons test	-	6	1.508	1.434	1.330	1.508	1.444	1.389
	Ch 1%	1.431	0.033		ns	6	1.522	1.360	1.524	1.412	1.438	1.328
	Ch 5%	1.458	0.023		ns	6	1.472	1.453	1.519	1.452	1.355	1.498
	Ch 10%	1.447	0.077		ns	6	1.682	1.347	1.560	1.221	1.592	1.280
	ChO 1%	1.423	0.026		ns	6	1.406	1.351	1.530	1.394	1.468	1.388
	ChO 5%	1.498	0.071		ns	6	1.684	1.732	1.297	1.451	1.357	1.467
	ChO 10%	1.396	0.016		ns	6	1.405	1.331	1.413	1.400	1.381	1.447
After the release study	Control	1.431	0.028		-	6	1.505	1.430	1.324	1.503	1.439	1.388
	Ch 1%	1.420	0.033		ns	6	1.510	1.350	1.514	1.400	1.429	1.316
	Ch 5%	1.444	0.023		ns	6	1.457	1.439	1.507	1.439	1.341	1.479
	Ch 10%	1.454	0.084		ns	6	1.669	1.337	1.549	1.213	1.684	1.270
	ChO 1%	1.416	0.026		ns	6	1.402	1.344	1.522	1.385	1.462	1.383
	ChO 5%	1.484	0.072		ns	6	1.671	1.720	1.285	1.435	1.340	1.454
	ChO 10%	1.375	0.016		ns	6	1.386	1.310	1.390	1.359	1.378	1.428
**Figure 4:** Cumulative release of vancomycin (μg) from bone cement prepared with chitosan (A) and chitosan oligosaccharides (B)												
Control	1	634.1	58.4	Two-way ANOVA with Dunnett's multiple comparisons test	-	6	497.8	649.9	658.1	440.3	818.4	740.3
	3	1186.0	95.0		-	6	1065.1	1272.1	1025.8	890.6	1516.0	1349.1
	6	1771.0	143.5		-	6	1501.1	1656.9	1737.0	1781.5	2448.1	1502.4
	24	2672.0	221.5		-	6	2439.1	2487.7	2610.7	2745.0	3679.1	2071.8
	48	2929.0	204.2		-	6	2502.5	2941.0	3392.2	3075.6	3470.8	2194.8
	72	3210.0	209.4		-	6	2876.1	2970.1	3732.5	3405.6	3780.0	2495.6
	120	2967.0	205.8		-	6	2559.2	2832.9	3493.4	3086.5	3542.7	2286.1
	168	3122.0	215.7		-	6	2590.7	3013.6	3610.1	3200.0	3813.8	2506.8

Figure number/Sample name (group)	Sample name (subgroup)	Mean	SEM	Statistical method used	P-value	Sample size (n)	Raw data					
Ch 1%	1	1123.0	170.1		ns	6	670.4	580.0	1201.1	1644.9	1242.2	1398.4
	3	1430.0	52.1		ns	6	1461.8	1294.4	1389.1	1395.7	1374.0	1666.9
	6	1651.0	91.9		ns	6	2073.5	1471.4	1446.6	1637.1	1623.4	1654.2
	24	2390.0	165.3		ns	6	2684.3	2304.0	2053.6	2250.0	1996.3	3051.6
	48	2731.0	223.7		ns	6	3559.7	2584.6	2207.5	2514.3	2274.4	3243.9
	72	3043.0	242.3		ns	6	3951.4	2773.8	2477.2	2886.8	2577.3	3593.0
	120	2836.0	206.5		ns	6	3573.9	2562.3	2354.3	2769.4	2420.5	3337.0
	168	3024.0	213.7		ns	6	3776.7	2749.1	2506.3	3004.2	2574.4	3531.8
Ch 5%	1	1025.0	110.8		ns	6	1307.9	851.2	1023.8	621.1	1012.1	1332.6
	3	1546.0	92.9		ns	6	1762.5	1500.7	1391.4	1197.1	1645.8	1779.8
	6	1910.0	113.2		ns	6	1928.1	1808.7	1986.6	1429.5	2058.7	2249.5
	24	2725.0	76.7		ns	6	2917.0	2634.4	2466.1	2604.8	2940.4	2785.0
	48	3205.0	158.0		ns	6	3295.2	2886.0	2965.8	2918.7	3256.5	3910.2
	72	3666.0	166.9		ns	6	3786.2	3380.1	3478.1	3274.7	3668.5	4408.5
	120	3291.0	191.0		ns	6	3067.0	3021.6	3128.0	2991.5	3323.0	4215.5
	168	3605.0	206.1		ns	6	3817.2	3320.4	3289.0	3205.6	3460.9	4536.5
Ch 10%	1	610.1	61.6		ns	6	908.8	543.0	604.7	493.7	571.8	538.9
	3	1238.0	80.9		ns	6	1568.4	1227.4	1344.5	1135.4	1154.4	997.1
	6	1623.0	90.3		ns	6	1595.2	1476.0	2067.5	1543.4	1522.5	1532.2
	24	2471.0	144.1		ns	6	2263.2	2588.9	3094.3	2106.6	2509.0	2263.2
	48	3119.0	179.7		ns	6	3696.6	3027.4	3614.3	2635.1	2978.1	2761.7
	72	3513.0	148.8		ns	6	3922.1	3277.9	4038.8	3293.9	3289.3	3253.2
	120	3321.0	213.8		ns	6	4025.5	3121.7	3938.8	2796.1	3005.2	3040.6
	168	3565.0	200.5		ns	6	4342.7	3297.7	3969.9	3046.9	3459.8	3271.2
ChO 1%	1	773.9	88.5	Two-way ANOVA with	ns	6	522.5	764.9	1160.0	666.3	838.9	691.0
	3	1380.0	102.2		ns	6	1128.1	1185.5	1461.7	1790.4	1492.1	1224.1

Figure number/Sample name (group)	Sample name (subgroup)	Mean	SEM	Statistical method used	P-value	Sample size (n)	Raw data					
	6	1833.0	94.2	Dunnett's multiple comparisons test	ns	6	1600.0	1753.8	1831.7	2276.4	1750.4	1784.7
	24	2384.0	162.6		ns	6	1949.2	2139.7	2883.5	2877.5	2247.8	2203.7
	48	3335.0	230.4		ns	6	2476.5	3291.7	4074.6	3723.0	3478.4	2966.5
	72	3710.0	304.3		ns	6	2756.1	3660.1	4857.1	4137.7	3743.4	3105.8
	120	3354.0	248.9		ns	6	2474.0	3396.5	4127.5	3790.4	3504.3	2833.0
	168	3283.0	265.0		ns	6	2122.0	3736.3	3655.0	3561.7	3719.8	2900.8
ChO 5%	1	544.4	77.5		ns	6	333.4	493.7	633.4	875.9	427.9	501.9
	3	1166.0	97.2		ns	6	862.3	1143.6	992.1	1559.1	1223.6	1217.8
	6	1427.0	100.9		ns	6	1217.1	1327.6	1327.1	1913.8	1416.2	1362.4
	24	2570.0	205.5		ns	6	3057.9	2531.7	1817.5	2144.5	2855.2	3015.5
	48	4204.0	200.6		<0.001	6	4671.6	4266.0	3350.7	3935.4	4436.0	4566.3
	72	5529.0	197.8		<0.001	6	6348.6	5493.2	4846.9	5636.2	5450.2	5397.6
	120	5438.0	198.7		<0.001	6	5723.8	5145.4	4732.8	6156.2	5388.5	5481.0
	168	5579.0	193.3		<0.001	6	5586.4	5187.7	5385.0	6344.4	5890.2	5081.9
ChO 10%	1	469.7	53.5		ns	6	456.7	440.3	337.5	481.4	715.6	386.8
	3	1007.0	101.1		ns	6	804.8	1388.4	907.7	731.7	1192.1	1016.7
	6	1692.0	178.6		ns	6	2316.7	1822.1	1642.7	977.5	1832.4	1560.0
	24	3220.0	300.3		ns	6	4027.8	3997.6	3489.7	2394.4	2989.0	2420.0
	48	4682.0	254.4		<0.001	6	4834.7	5084.8	4545.4	3626.5	5458.9	4543.1
	72	6518.0	354.6		<0.001	6	6296.7	7085.0	7464.0	5443.7	7223.2	5592.6
	120	6755.0	412.6		<0.001	6	5877.2	8509.8	6801.1	5886.6	7226.8	6227.4
	168	7018.0	134.6		<0.001	6	6646.1	7247.7	7064.6	7084.7	7447.1	6616.1

Figure 5: Cumulative release of gentamicin (μg) from bone cement prepared with chitosan (A) and chitosan oligosaccharides (B)

Control	1	55.9	18.1	Two-way ANOVA with Dunnett's	-	3	32.2	43.9	91.5
	3	74.9	23.3		-	3	56.1	47.4	121.1
	6	128.8	22.7		-	3	99.1	113.9	173.5

Figure number/Sample name (group)	Sample name (subgroup)	Mean	SEM	Statistical method used	P-value	Sample size (n)	Raw data		
	24	323.6	34.4	multiple comparisons test	-	3	305.4	390.2	275.2
	48	521.6	76.2		-	3	527.7	650.5	386.6
	72	795.4	155.2		-	3	804.3	1059.7	522.4
	120	1284.0	296.7		-	3	1396.1	1733.5	723.9
	168	1595.0	401.9		-	3	1550.3	2312.1	921.9
Ch 1%	1	93.6	14.9		ns	3	68.6	92.0	120.2
	3	124.3	0.4		ns	3	124.3	123.7	125.0
	6	178.9	8.8		ns	3	176.4	165.0	195.3
	24	403.6	20.9		ns	3	396.6	371.4	442.7
	48	616.4	36.3		ns	3	622.8	550.5	675.7
	72	914.9	80.0		ns	3	869.3	804.9	1070.6
	120	1359.0	93.3		ns	3	1314.0	1224.2	1538.1
	168	1693.0	100.8		ns	3	1682.2	1524.7	1873.3
Ch 5%	1	90.1	5.1		ns	3	80.0	94.3	96.1
	3	150.6	11.2		ns	3	131.1	150.8	169.7
	6	244.8	6.6		ns	3	232.4	255.0	247.0
	24	529.3	43.0		ns	3	482.8	489.9	615.1
	48	813.1	57.5		ns	3	734.4	779.9	925.1
	72	1165.0	29.0		ns	3	1118.6	1218.1	1157.1
	120	1641.0	52.2		ns	3	1537.6	1705.5	1679.6
	168	2030.0	56.1		<0.05	3	2099.6	2071.3	1918.8
Ch 10%	1	76.8	6.7		ns	3	71.0	90.2	69.3
	3	143.6	6.3		ns	3	133.2	154.8	142.9
	6	252.4	71.2		ns	3	203.2	392.7	161.4
	24	500.0	78.3		ns	3	533.6	615.6	350.7
	48	843.9	178.1		ns	3	751.6	1187.9	592.1

Figure number/Sample name (group)	Sample name (subgroup)	Mean	SEM	Statistical method used	P-value	Sample size (n)	Raw data		
	72	1105.0	132.6		ns	3	1117.1	1328.4	869.6
	120	1591.0	197.1		ns	3	1547.2	1952.7	1274.2
	168	2064.0	222.6		<0.05	3	1889.5	2506.1	1796.8
ChO 1%	1	67.3	6.5	Two-way ANOVA with Dunnett's multiple comparisons test	ns	3	59.5	80.3	62.2
	3	120.9	15.7		ns	3	95.1	118.5	149.2
	6	193.2	22.0		ns	3	166.2	176.7	236.7
	24	486.2	74.8		ns	3	347.0	508.4	603.3
	48	934.1	72.7		<0.05	3	791.7	1030.3	980.3
	72	1379.0	94.9		<0.01	3	1190.6	1494.9	1450.8
	120	2187.0	71.7		<0.001	3	2046.0	2279.2	2237.0
	168	2585.0	247.7		<0.001	3	2092.4	2879.2	2782.1
ChO 5%	1	68.1	8.2		ns	3	53.7	68.7	82.0
	3	103.1	16.6		ns	3	84.9	88.1	136.2
	6	160.9	29.2		ns	3	128.2	135.4	219.1
	24	364.2	43.5		ns	3	407.5	277.2	408.0
	48	820.6	78.2		ns	3	961.3	691.1	809.2
	72	1448.0	55.3		<0.001	3	1516.2	1339.0	1490.2
	120	2211.0	136.6		<0.001	3	2203.3	1978.3	2451.2
	168	2813.0	221.2		<0.001	3	2861.6	2408.1	3169.6
ChO 10%	1	39.1	1.8		ns	3	37.0	42.7	37.6
	3	57.3	5.6		ns	3	68.3	50.1	53.3
	6	95.0	2.3		ns	3	95.6	98.7	90.7
	24	325.1	33.3		ns	3	374.3	339.3	261.6
	48	659.2	21.5		ns	3	674.0	616.9	686.7
	72	1286.0	33.0		<0.05	3	1244.3	1262.2	1351.1
	120	2010.0	59.3		<0.001	3	1928.0	1977.8	2125.5

Figure number/Sample name (group)	Sample name (subgroup)	Mean	SEM	Statistical method used	P-value	Sample size (n)	Raw data					
	168	2726.0	48.3		<0.001	3	2629.3		2772.2		2776.1	
**Figure 6:** Zone of inhibition (ZOI, mm) of *S. aureus* (A) and MRSA (B) of the supernatant obtained from different bone cement specimens that incubated in PBS for 7 days												
S. *aureus*	Control	25.9	0.3	One-way ANOVA with Dunnett's multiple comparisons test	-	6	25.0	25.0	26.0	26.8	25.8	26.5
	Ch 1%	26.5	0.3		ns	6	25.5	26.0	26.0	27.0	27.3	27.2
	Ch 5%	27.1	0.2		<0.01	6	26.5	26.7	27.0	27.3	27.5	27.5
	Ch 10%	28.0	0.1		<0.001	6	27.5	28.0	28.2	27.8	28.1	28.5
	ChO 1%	27.0	0.1		<0.01	6	26.7	27.0	26.8	26.8	27.2	27.5
	ChO 5%	28.7	0.1		<0.001	6	28.5	29.0	29.0	28.5	28.5	28.8
	ChO 10%	29.0	0.2		<0.001	6	28.5	29.0	30.0	28.8	28.9	28.9
MRSA	Control	10.9	0.3	One-way ANOVA with Dunnett's multiple comparisons test	-	6	10.1	10.3	10.1	11.6	11.8	11.4
	Ch 1%	11.2	0.4		ns	6	10.2	10.3	10.4	12.3	11.9	12.1
	Ch 5%	11.7	0.5		ns	6	10.9	10.5	10.7	12.5	12.9	12.8
	Ch 10%	12.4	0.4		<0.05	6	11.4	11.6	11.5	13.2	13.7	13.0
	ChO 1%	11.3	0.3		ns	6	10.6	10.7	10.6	12.1	12.0	11.9
	ChO 5%	13.7	0.4		<0.001	6	12.8	12.9	12.8	14.7	14.3	14.4
	ChO 10%	14.1	0.3		<0.001	6	13.3	13.6	13.4	14.9	15.1	14.3
**Figure 7:** Relative cell viability (%) of Saos-2 cells after treatment with extracts from various bone cement samples for 24 h												
PBS-treated	-	100.00	1.13	Not applicable	-	41	109.01	106.76	113.06		106.31	105.41
							99.56	93.26	100.91	104.06	109.01	104.96
							94.16	103.61	96.41	99.56	102.26	95.96
							110.81	100.46	98.21	112.16	103.16	107.66
							91.46	100.01	98.66	104.51	107.66	108.11
							90.56	87.41	91.46	102.26	94.16	89.21
							88.31	86.51	90.11	100.91	95.51	96.41
Control	-	108.60	0.85		-	36	102.26	104.06	105.41	104.06	107.66	103.61

Figure number/Sample name (group)	Sample name (subgroup)	Mean	SEM	Statistical method used	P-value	Sample size (n)	Raw data					
							101.36	104.06	110.36	106.31	106.76	101.36
							103.16	108.11	107.21	101.36	114.42	104.06
							105.41	110.36	116.22	113.97	112.16	111.71
							111.26	120.72	111.71	106.31	114.42	101.81
							114.87	113.97	116.22	109.91	111.71	110.36
Ch 1%	-	98.19	0.76		-	36	89.66	98.66	93.71	94.61	95.51	93.71
							97.31	90.11	100.46	102.26	94.61	93.71
							95.51	94.61	102.71	97.76	96.86	92.81
							95.06	96.41	101.81	104.06	95.06	94.16
							100.91	99.56	108.11	107.21	105.86	98.66
							101.81	102.71	102.71	102.26	97.31	96.41
Ch 5%	-	96.65	0.45		-	36	99.56	99.11	97.76	96.41	97.31	91.91
							100.46	99.56	97.31	93.26	94.16	96.86
							95.96	95.06	104.51	100.46	100.46	95.51
							92.36	95.06	95.96	97.31	95.96	92.36
							94.16	96.41	94.16	95.06	98.66	97.76
							95.06	100.01	95.06	94.61	98.21	95.51
Ch 10%	-	107.60	0.70		-	36	106.76	112.16	112.16	115.77	104.51	105.41
							99.11	103.16	109.46	107.21	107.21	109.01
							104.06	108.11	110.36	113.97	111.71	109.91
							102.71	99.11	109.46	112.16	106.76	100.46
							101.36	106.31	111.26	110.81	106.76	109.46
							101.36	110.81	110.81	111.26	106.76	105.41
ChO 1%	-	101.60	0.72		-	36	98.21	95.96	104.96	101.36	98.21	103.16
							94.61	103.16	103.16	105.41	94.61	99.11
							106.31	106.31	105.41	105.86	106.76	95.51

Figure number/Sample name (group)	Sample name (subgroup)	Mean	SEM	Statistical method used	P-value	Sample size (n)	Raw data					
							102.26	104.96	107.21	106.76	103.16	103.61
							94.16	97.31	96.86	100.01	97.31	95.06
							106.76	103.16	109.01	99.56	100.91	100.01
ChO 5%	-	89.40	0.51		-	36	88.76	87.86	92.36	89.66	91.46	93.71
							84.71	85.61	83.36	89.21	85.16	87.41
							85.16	90.56	86.96	86.06	89.21	88.76
							86.96	87.41	89.66	87.41	91.01	92.81
							88.31	87.86	90.56	91.01	95.96	96.41
							88.76	89.66	94.16	90.11	92.36	91.91
ChO 10%	-	87.16	0.43		-	36	89.21	86.96	86.51	81.56	85.16	87.86
							86.96	86.06	87.41	81.56	82.91	86.51
							84.26	91.01	85.16	86.51	88.31	89.66
							85.61	82.91	87.41	86.51	86.96	90.56
							89.21	86.06	92.36	85.16	86.51	91.01
							89.66	90.56	89.66	88.31	87.86	87.86
**Figure 8A:** Roughness (μm) of various bone cement samples before and after the drug release study												
Before the release study	Control	5.72	0.79	Two-way ANOVA with Dunnett's multiple comparisons test	-	5	5.66	6.65	3.80	4.33	8.15	
	Ch 1%	8.47	0.76		ns	5	10.63	9.10	5.93	8.08	8.61	
	Ch 5%	7.49	0.76		ns	5	7.70	7.93	6.87	9.79	5.14	
	Ch 10%	9.66	2.23		<0.01	5	6.34	18.17	10.03	7.29	6.47	
	ChO 1%	5.89	1.15		ns	5	3.90	5.29	6.02	4.07	10.20	
	ChO 5%	5.78	0.99		ns	5	6.36	4.12	4.21	4.85	9.38	
	ChO 10%	5.62	0.53		ns	5	5.56	4.35	5.23	7.57	5.37	
After the release study	Control	5.40	0.51	Two-way ANOVA with Dunnett's	-	6	5.66	6.65	3.80	4.33	6.94	4.99
	Ch 1%	6.05	0.27		ns	6	6.00	6.74	5.27	5.65	5.65	6.95
	Ch 5%	6.35	0.61		ns	6	6.41	4.58	8.23	7.43	6.86	4.58

Figure number/Sample name (group)	Sample name (subgroup)	Mean	SEM	Statistical method used	P-value	Sample size (n)	Raw data					
	Ch 10%	7.85	0.53	multiple comparisons test	ns	6	9.33	6.00	8.89	8.25	8.05	6.57
	ChO 1%	4.55	0.22		ns	6	4.23	4.26	4.87	5.42	4.62	3.88
	ChO 5%	4.66	0.21		ns	6	4.98	4.92	3.96	4.92	5.14	4.03
	ChO 10%	5.38	0.20		ns	5	5.27	5.06	5.54	6.06	4.98	
**Figure 8B:** Microhardness (HV) of various bone cement samples before and after the drug release study												
Before the release study	Control	15.49	0.44	Two-way ANOVA with Dunnett's multiple comparisons test comparing to the control group	-	5	14.65	15.57	14.37	16.22	16.64	
	Ch 1%	16.60	0.56		<0.05	5	15.82	15.32	16.57	16.64	18.63	
	Ch 5%	17.08	0.25		<0.001	5	17.52	17.29	16.85	16.22	17.52	
	Ch 10%	15.51	0.43		ns	5	15.19	16.43	16.64	14.54	14.77	
	ChO 1%	16.90	0.32		<0.01	5	16.64	17.22	16.57	17.98	16.09	
	ChO 5%	17.32	0.25		<0.001	5	17.59	16.71	16.92	18.14	17.22	
	ChO 10%	16.09	0.26		ns	5	16.22	15.89	16.85	16.22	15.25	
After the release study	Control	13.77	0.09	Two-way ANOVA with Dunnett's multiple comparisons test comparing to the control group	-	6	13.76	13.81	14.06	13.58	13.47	13.93
	Ch 1%	14.67	0.11		<0.05	6	15.04	14.58	14.38	14.57	14.96	14.52
	Ch 5%	14.48	0.13		ns	6	14.55	13.98	14.91	14.70	14.47	14.25
	Ch 10%	13.30	0.06		ns	6	13.33	13.06	13.51	13.22	13.30	13.38
	ChO 1%	14.35	0.11		ns	6	14.36	14.09	14.17	14.54	14.20	14.77
	ChO 5%	14.29	0.09		ns	6	14.47	14.01	14.39	14.17	14.59	14.11
	ChO 10%	12.85	0.14		<0.05	6	12.83	12.20	12.87	13.00	13.14	13.05
**Supplementary Figure 1A:** Diameter (mm) of bone cement samples obtained after casting bone cement mixtures in the mold.												
Control	-	18.38	0.14		-	12	18.00	18.00	18.00	19.00	18.00	18.00

Figure number/Sample name (group)	Sample name (subgroup)	Mean	SEM	Statistical method used	P-value	Sample size (n)	Raw data					
							19.00	18.20	19.00	19.00	18.00	18.40
Ch 1%	-	18.37	0.10	One-way ANOVA with Dunnett's multiple comparisons test	ns	12	18.05<br>19.00	18.20<br>18.30	18.30<br>18.00	18.50<br>19.00	18.30<br>18.55	18.20<br>18.00
Ch 5%	-	18.33	0.11		ns	12	18.05<br>18.30	18.30<br>18.40	18.30<br>18.00	18.00<br>18.60	19.00<br>18.00	18.00<br>19.00
Ch 10%	-	18.63	0.15		ns	12	18.10<br>19.00	19.00<br>19.00	18.00<br>18.45	18.50<br>19.00	19.55<br>19.00	18.00<br>18.00
ChO 1%	-	18.18	0.09		ns	12	18.00<br>19.00	18.00<br>18.00	18.20<br>18.20	18.00<br>18.40	18.00<br>18.00	18.00<br>18.40
ChO 5%	-	18.14	0.17		ns	12	18.30<br>18.30	18.10<br>18.40	18.00<br>17.00	19.00<br>19.00	18.00<br>18.00	17.30<br>18.30
ChO 10%	-	18.35	0.16		ns	12	19.00<br>18.50	19.00<br>18.10	19.00<br>18.40	18.00<br>18.00	18.00<br>19.00	17.20<br>18.00
**Supplementary Figure 1B:** Thickness (mm) of bone cement samples obtained after casting bone cement mixtures in the mold.												
Control	-	5.38	0.16	One-way ANOVA with Dunnett's multiple comparisons test	-	12	6.00<br>5.00	5.00<br>5.00	5.00<br>5.00	6.00<br>6.00	6.00<br>6.00	4.50<br>5.00
Ch 1%	-	5.31	0.13		ns	12	5.00<br>5.00	5.00<br>5.00	6.00<br>6.00	6.00<br>5.00	5.30<br>5.40	5.00<br>5.00
Ch 5%	-	5.23	0.09		ns	12	5.20<br>5.30	5.40<br>5.00	5.00<br>6.00	5.00<br>5.00	5.10<br>5.00	5.50<br>5.20
Ch 10%	-	5.48	0.16		ns	12	6.00<br>6.00	6.00<br>5.00	5.00<br>5.25	6.40<br>5.00	5.10<br>6.00	5.00<br>5.00
ChO 1%	-	5.09	0.17		ns	12	6.00<br>5.00	5.00<br>5.00	4.00<br>5.60	5.10<br>5.00	6.00<br>5.00	5.00<br>4.40
ChO 5%	-	5.44	0.14		ns	12	5.40<br>6.00	5.30<br>6.00	5.30<br>5.00	6.00<br>5.00	6.00<br>5.00	4.60<br>5.70

Figure number/Sample name (group)	Sample name (subgroup)	Mean	SEM	Statistical method used	P-value	Sample size (n)	Raw data					
ChO 10%	-	5.02	0.17		ns	12	5.00	5.00	5.00	5.00	5.00	4.00
							5.00	4.20	5.00	6.00	5.00	6.00
**Supplementary Figure 4:** Relative cell viability (%) of Saos-2 cells after treatment with various concentration of SLS (25 – 100 μg/mL) for 24 h												
PBS-treated	-	100.00	1.13	Not applicable	-	41	109.01	106.76	113.06		106.31	105.41
							99.56	93.26	100.91	104.06	109.01	104.96
							94.16	103.61	96.41	99.56	102.26	95.96
							110.81	100.46	98.21	112.16	103.16	107.66
							91.46	100.01	98.66	104.51	107.66	108.11
							90.56	87.41	91.46	102.26	94.16	89.21
							88.31	86.51	90.11	100.91	95.51	96.41
SLS 25 μg/mL	-	94.85	1.67		-	21	109.46	114.87	104.96	87.41	91.91	95.51
							86.51	91.91	95.51	94.61	101.81	100.01
							97.31	89.21	91.01	86.51	91.01	93.26
							92.36	86.96	89.66			
SLS 50 μg/mL	-	66.77	1.95		-	21	86.06	74.81	70.75	76.16	71.65	67.15
							63.55	71.20	68.95	76.61	72.10	70.75
							70.75	56.80	57.70	61.75	62.65	64.90
							55.00	52.75	50.05			
SLS 75 μg/mL	-	11.81	1.14		-	21	22.59	15.84	12.24	11.79	11.79	10.44
							5.04	5.94	5.04	10.89	7.74	8.64
							11.34	10.89	13.59	8.19	9.54	7.29
							22.59	21.24	15.39			
SLS 100 μg/mL	-	6.34	0.65		-	21	5.49	5.49	5.49	5.04	4.59	4.59
							4.14	4.14	3.24	5.94	5.49	5.94
							5.49	5.04	7.29	5.94	5.49	5.04

Figure number/Sample name (group)	Sample name (subgroup)	Mean	SEM	Statistical method used	P-value	Sample size (n)	Raw data					
							13.14	13.14	13.14			
untreated	-	105.20	1.26		-	42	116.67	119.37	121.17	113.51	118.02	114.42
							105.41	111.26	107.66	108.56	104.96	102.26
							103.16	104.96	105.86	105.41	105.86	109.91
							113.97	111.71	113.06	111.26	113.97	102.71
							100.91	100.01	97.31	110.81	109.46	104.51
							90.56	91.91	90.56	97.76	97.76	98.21
							91.46	92.81	92.81	103.16	99.56	104.51

*ns: not significant
