# Supplementary material for: Influence of chitosan and chitosan oligosaccharide on dual antibiotic-loaded bone cement: In vitro evaluations
Source: PLoS One. 2022 Nov 30;17(11):e0276604. doi: 10.1371/journal.pone.0276604 (PMC9710798; doi:10.1371/journal.pone.0276604)
Supplement: Attachment — Submitted filename: review comments.docx [file pone.0276604.s006.docx]

The submitted work is interesting as the Author not only presented their work but has highlighted the limitations of the study. A few minor revisions were suggested.

1. Language and grammar need to be improved. Many sentences are missing adjectives, and articles are not properly formatted, making them difficult to understand. Also, a few references cited in the text are incomplete.
2. The following sentences seem repetitive. Authors are kindly advised to improve the sentence structure to avoid confusion and improve readability.

*Polymethylmethacrylate (PMMA) bone cement has many important roles in orthopaedics. It’s also played an imminent role in the treatment of orthopaedic infection.*

*In the current practice, ALBC is used as a prophylaxis to prevent prosthetic joint infections (PJIs) in total joint arthroplasty. Moreover, ALBC is also used to treat orthopaedic infections such as PJIs and chronic osteomyelitis.*

3. “To prepare the Ch and the ChO mixed bone cement samples, different amounts of Ch and ChO were added to the polymethylmethacrylate matrix with three concentrations (1%, 5%, and 10%)”. Authors kindly specify the basis for the selection of the aforementioned conditions. Also, comment on why only three concentrations were taken.
4. As mentioned in the introduction section manual mixing has several limitations. Still, the technique followed for the present work is manual mixing. Authors kindly justify why this is selected.
5. For antibacterial activity why only *S. aureus* and MRSA were considered. Are they predominant causative agents for orthopaedic infection? Authors kindly comment.
6. Why is it preferable to use a pre-warmed PBS for drug elution assay?
